# Detecting Abnormal Brain Regions in Schizophrenia Using Structural MRI via Machine Learning

**DOI:** 10.1155/2020/6405930

**Published:** 2020-04-05

**Authors:** ZhiHong Chen, Tao Yan, ErLei Wang, Hong Jiang, YiQian Tang, Xi Yu, Jian Zhang, Chang Liu

**Affiliations:** ^1^College of Information Technology and Engineering, Chengdu University, Chengdu, China; ^2^Key Laboratory of Pattern Recognition and Intelligent Information Processing in Sichuan, Chengdu, China; ^3^Department of Radiology, The Second Affiliated Hospital of Soochow University, Suzhou, China; ^4^Department of Neurosurgery, Rui-Jin Hospital, Shanghai Jiao Tong University School of Medicine, Shanghai, China; ^5^School of Physics and Electronic Engineering, Sichuan Normal University, Chengdu, China; ^6^The Clinical Hospital of Chengdu Brain Science Institute, MOE Key Lab for Neuroinformation, Center for Information in Medicine, University of Electronic Science and Technology of China, Chengdu, China; ^7^School of Life Science and Technology, University of Electronic Science and Technology of China, Chengdu, China; ^8^College of Computer Science, Sichuan Normal University, Chengdu, Sichuan, China

## Abstract

Utilizing neuroimaging and machine learning (ML) to differentiate schizophrenia (SZ) patients from normal controls (NCs) and for detecting abnormal brain regions in schizophrenia has several benefits and can provide a reference for the clinical diagnosis of schizophrenia. In this study, structural magnetic resonance images (sMRIs) from SZ patients and NCs were used for discriminative analysis. This study proposed an ML framework based on coarse-to-fine feature selection. The proposed framework used two-sample *t*-tests to extract the differences between groups first, then further eliminated the nonrelevant and redundant features with recursive feature elimination (RFE), and finally utilized the support vector machine (SVM) to learn the decision models with selected gray matter (GM) and white matter (WM) features. Previous studies have tended to report differences at the group level instead of at the individual level and cannot be widely applied. The method proposed in this study extends the diagnosis to the individual level and has a higher recognition rate than previous methods. The experimental results of this study demonstrate that the proposed framework distinguishes SZ patients from NCs, with the highest classification accuracy reaching over 85%. The identified biomarkers are also consistent with previous literature findings. As a universal method, the proposed framework can be extended to diagnose other diseases.

## 1. Introduction

Schizophrenia (SZ) is a group of major psychiatric diseases with unknown etiology. SZ has the highest prevalence of all mental illnesses and is very difficult to treat. Over the last few decades, many neuroimaging studies have demonstrated that schizophrenia is a disorder involving widespread abnormalities in the brain structure [1–5]. However, the specific mechanisms involved in producing these structural deficits remain incompletely understood. In recent years, it has been consistently reported that SZ patients have structural abnormalities in the brain, including the middle temporal gyrus, middle frontal gyrus, thalamus, and corpus callosum (CcSum) [6–8]. The brain structure location and neurobiological processes underlying these structural abnormalities are central to the pathophysiology of schizophrenia. Furthermore, alterations to the brain structure are linked to key psychotic symptoms (such as auditory hallucinations [9, 10], neurosensory deficits [11, 12], and social dysfunction [13, 14] in SZ).

At present, the diagnosis and monitoring of SZ mainly hinge on doctors' judgment through patients' clinical response, history, and neurological examination. The diagnosis and monitoring of SZ are heavily dependent on doctors' clinical experience and related knowledge. In other words, this subjective judgment may add risk to the diagnosis and treatment of SZ. For a more accurate diagnosis, neuroimaging methods have been widely used to study brain morphology, which provides important information about possible pathophysiologic mechanisms [15–18].

Due to its good contrast and high spatial resolution, structural magnetic resonance imaging (sMRI) has become one of the most popular neuroimaging modalities [19–21]. Most existing research has investigated conventional statistical analysis methods to explore the differences between SZ patients and normal controls (NCs) based on group studies [15, 22, 23]. Despite the ability of conventional statistical analysis methods to detect some abnormal brain regions in SZ, they are univariate methods and often overlook the correlations among voxels, which often contain important characteristic information. Furthermore, conventional statistical analysis only considers differences among groups, and it is difficult to generalize the diagnosis to individual patients.

To overcome the drawbacks of conventional statistical analysis, machine learning (ML) techniques have been applied to analyze neuroimaging data. These techniques can extract stable structural or functional patterns from neuroimaging data and may potentially be useful for finding significant neuroimaging-based biomarkers. Currently, promising results have been reported for the classification of SZ patients and NCs [24–26].

The most common feature of sMRI is the so-called brain tissue volume (obtained from voxel-based morphometry). However, the existence of too many irrelevant features can greatly degrade the classification accuracy, especially in neuroimaging studies. The preprocessed brain MRI may contain >100,000 nonzero voxels. In comparison, the sample size (number of subjects or observations) is often less than 1000 [27]. Thus, the number of features (voxels) greatly exceeds the number of observations (sample size). This issue is a common problem in machine learning studies and is known as the “curse of dimensionality” [28–30]. The curse of dimensionality can lead to overfitting of the learned model. Therefore, choosing and utilizing appropriate feature selection methods can effectively improve the performance of the model.

For most supervised ML studies, the corresponding supervised feature selection method uses high-dimensional neuroimaging data and the required outcome labels (e.g., +1 treatment responders and −1 treatment nonresponders) to select relevant features and discard redundant features and noise [27]. More specifically, these techniques are subdivided into three categories [30–32]: (1) “filter methods,” which use simple statistical measures (e.g., mean, variance, and correlation coefficients) to rank features according to their relevance in detecting group-level differences, such as s *t*-tests, analysis of variance (ANOVA), and Pearson correlation coefficients; (2) “wrapper methods,” which use a cost function to optimize the machine learning model and rank features in terms of their relevance; and (3) “embedded methods,” which select relevant features as “part” of the machine learning process by enforcing certain “penalties” on the machine learning model to yield a small subset of relevant features.

A recent study [33] utilized a support vector machine (SVM) to learn the decision model to classify Alzheimer's disease patients and normal controls, which achieved an area under the curve (AUC) of over 88.82%. Another study [8] used cortical thickness in conjunction with surface area in schizophrenia patients to perform discriminative analysis and obtained an accuracy of 85.0%. Though the good performance with machine learning produced an excessive number of features (voxels), there is a risk of overfitting [34, 35]. To overcome this problem, a large number of feature selection methods have been proposed. One study [36] selected discriminative features using Fisher's criterion to train the SVM model. As a result, the classification accuracy reached 76.25% for identifying bipolar disorder patients from normal controls. Another study [37] detected first episode psychosis, which utilized principal component analysis (PCA) to reduce the number of nonrelevant features in cortical thickness and gray matter volume and then applied deep neural networks (DNNs) to construct the classification model. The authors achieved a classification accuracy of over 70.5%. However, most of these studies have analyzed only gray matter (GM). In fact, several studies [17, 38, 39] have demonstrated that there is a nonnegligible change in white matter (WM) in SZ and that it is also necessary to analyze WM.

As an effective feature selection algorithm, recursive feature elimination (RFE) evaluates the contribution of each feature and then eliminates the smallest contribution features iteratively [40–42]. In this study, a machine learning framework based on coarse-to-fine feature selection is proposed. The framework first uses two-sample *t*-tests to roughly select features and then eliminates the nonrelevant and redundant features via RFE. Finally, the SVM is utilized to learn the decision models for WM and GM separately. The experimental results demonstrate that the proposed method is able to differentiate SZ patients from NCs with a maximum accuracy of approximately 85% and can find biomarkers of SZ that are consistent with those found in previous studies, including the left and right middle temporal gyrus, right middle frontal gyrus, thalamus, corpus callosum, fusiform gyrus, occipital lobe, cuneus, postcentral gyrus, and cerebellum.

The contributions of our work include the following:

1. We developed a machine learning framework to differentiate SZ patients from NCs. The proposed machine learning framework adopts a coarse-to-fine approach to roughly reduce the dimensionality of features with two-sample *t*-tests and then further with RFE. Hierarchical feature selection is helpful for preserving informative features and eliminating redundant ones. Furthermore, coarse-to-fine feature selection is easy to use to identify biomarkers of schizophrenia (abnormal brain regions). The proposed machine learning framework does not apply to schizophrenia only. It can also be generalized to other diseases to classify patients and NCs based on sMRI.
2. The experimental results demonstrate that the proposed method achieves a better classification performance than other methods. Furthermore, the identified biomarkers are consistent with the findings of previous related research works.
3. Previous research works have mainly focused on gray matter and have seldom investigated white matter in schizophrenia patients. This study analyzes gray matter and white matter separately and finds that white matter has a better discriminative ability than gray matter, which provides a reference for clinical diagnosis.

## 2. Materials and Methods

### 2.1. Subjects and MRI Data Acquisition

The imaging data and phenotypic information used in this study were obtained from the Centers for Biomedical Research Excellence (COBRE) dataset, which was collected and shared by the Mind Research Network and The University of New Mexico (http://fcon_1000.projects.nitrc.org/indi/retro/cobre.html). To reduce the impact of different subtypes of SZ, we chose only paranoid schizophrenia from the dataset. Paranoid schizophrenia is the most common type of SZ and has a slower course of disease development, a later neurodegenerative onset time, and a better curative effect [43, 44].

In this study, we selected 34 paranoid schizophrenia patients and 34 normal controls from the dataset. The selected subjects were right-handed and were aged between 20 and 60. All subjects were examined and excluded if they had a history of a neurological disorder, a history of mental retardation, a history of severe head trauma with more than 5 minutes of loss of consciousness, or a history of substance abuse or dependence within the last 12 months. Diagnostic information was gathered using the Structured Clinical Interview for DSM Disorders (SCID). The demographics are reported in Table 1.

All sMRI data were acquired with a multiecho MPRAGE (MEMPR) sequence. The parameters used were a repetition time (TR) of 2530 ms; echo times (TEs) of 1.64, 3.5, 5.36, 7.22, and 9.08 ms; an inversion time (TI) of 900 ms; an FOV (field of view) of 256 × 256 mm; a matrix of 256 × 256 × 176; a flip angle of 7°; a voxel size of 1 × 1 × 1 mm; a slab thickness of 176 mm; a number of echoes of 5; and a total scan time of 6 min.

### 2.2. Preprocessing

sMRI data were analyzed with the Statistical Parametric Mapping (SPM) software package SPM8 (Wellcome Department of Imaging Neuroscience, London, UK; http://www.fil.ion.ucl.ac.uk/spm) using the voxel-based morphometry (VBM) [45, 46] protocol. First, spatial normalization of all 3D volumes with the T1 template was provided by SPM8 for bias correction (removal of positional and volume differences). Second, each T1-weighted MRI was segmented into three tissue probability maps (TPMs), including GM, WM, and cerebrospinal fluid (CSF). Third, the tissue volume was obtained by modulating the segmented tissue maps. Finally, a Gaussian kernel with a 6 mm isotropic full width at half maximum was employed for spatial smoothing.

### 2.3. Machine Learning Framework

**Figure 1:**
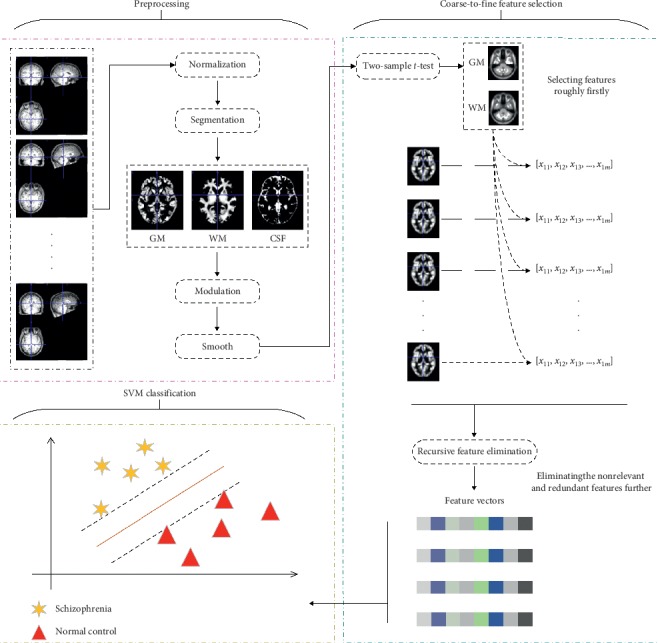
Workflow diagram of the proposed framework. The framework contains three phases: (1) the preprocessing phase, which normalizes the brains of different subjects into the standard MNI space, segments them into gray matter (GM), white matter (WM), and cerebrospinal fluid (CSF), and smoothes voxels to reduce the effects of noise; (2) the feature selection phase, which uses two-sample *t*-tests and RFE to select discriminative features with a coarse-to-fine approach; and (3) the feature classification phase, which employs the linear SVM to classify features.

After the preprocessing step, feature selection based on the coarse-to-fine approach was conducted to reduce the dimensionality of the features. First, two-sample *t*-tests were conducted to roughly select features, and then, RFE was used to further eliminate nonrelevant and redundant features. Lastly, a linear SVM classifier was trained to classify SZ patients and NCs. The workflow of the proposed machine learning framework is shown in Figure 1.

### 2.4. Feature Selection

To obtain a good classification performance, two-sample *t*-tests were used to perform a rough preliminary selection in this paper. Then, RFE was used to further select discriminative features.

#### 2.4.1. Two-Sample *t*-Tests

Due to the large amount of redundant information in sMRI, two-sample *t*-tests were used to initially screen the voxels. As a classical statistical analysis method, two-sample *t*-tests can extract significant differences between groups by computing the statistical significance value. Suppose ${\bar{x}}_{1}$ and ${\bar{x}}_{2}$ represent the means of a feature of the two groups. *S*_1_^2^ and *S*_2_^2^ denote the corresponding variances, and the significant differences between groups on this feature can be calculated as follows:

(1) $$\begin{array}{c} T=\frac{\left|{\bar{x}}_{1}-{\bar{x}}_{2}\right|}{\sqrt{\left(\left(\left(N_{1}-1\right)S_{1}^{2}+\left(N_{2}-1\right)S_{2}^{2}\right)/\left(N_{1}+N_{2}-2\right)\right)\cdot \left(\left(1/N_{1}\right)+\left(1/N_{2}\right)\right)}}, \end{array}$$

where *N*_1_ and *N*_2_ denote the sample sizes. The ability of a feature to distinguish between two groups is evaluated by the absolute value of *T*. The greater the absolute value of *T*, the more discriminative the feature.

#### 2.4.2. Recursive Feature Elimination

RFE [47] is a greedy method for ranking all features to obtain an optimal feature subset for classification. To perform this ranking, RFE trains a machine learning model (e.g., linear support vector machine or relevance vector machine), then ranks all features in terms of some specific ranking criteria, and finally removes the features with the smallest rankings. The procedure is repeated until all features are removed. Since RFE can eliminate a fixed quantity or percentage of features depending on the user's requirements and has a strong ability to explain differences, it has been popular in neuroimaging studies [40–42].

Currently, most studies [48–51] have combined RFE with the SVM to perform feature selection. The SVM is presently one of the best-known classification techniques and has computational advantages over other classification methods, and many previous studies [52–56] have proven that the linear SVM performs well in small sample datasets. To allow the classifier to generalize unseen data well and to avoid overfitting problems, we introduced the SVM soft margin classifier.

Taking the soft margin SVM as an example, assuming *m* input training samples *x*={*x*_1_, *x*_2_,…, *x*_*m*_}, each sample has *n* features (i.e., *x*_*i*_={*x*_*i*1_, *x*_*i*2_,…, *x*_in_}, *i*={1,2,…, *m*}) and corresponding labels *y*={*y*_1_, *y*_2_,…, *y*_*m*_}; the decision function *D*(*x*) is formulated as

(2) $$\begin{array}{c} \begin{array}{c} D\left(x\right)=\underset{\omega ,b,\zeta_{i}}{\min}\frac{1}{2}{\left\|\omega \right\|}^{2}+C\sum_{i=1}^{m}\zeta_{i},\\ \text{precondition}:\\ \zeta_{i}=1-y_{i}\left(\omega^{T}x_{i}+b\right)\\ \text{subject to}\\ \begin{array}{c} y_{i}\left(\omega^{T}x_{i}+b\right)\geq 1-\zeta_{i},\\ \zeta_{i}\geq 0, \end{array} \end{array} \end{array}$$

where *ω* is the weights of features, *C* is a nonzero penalty coefficient that controls the trade-off between the training error and the margin, and *ζ* is called slack variables that are associated with the misclassified samples.

Since the above optimization problem is difficult to solve, it can be rewritten as a dual problem using a Lagrangian multiplier method as follows:

(3) $$\begin{array}{c} \begin{array}{c} D\left(x\right)=\underset{a}{\max}\sum_{i=1}^{m}\alpha_{i}-\frac{1}{2}\sum_{i,j=1}^{m}\alpha_{i}\alpha_{j}y_{i}y_{j}x_{i}^{T}x_{j},\\ \text{subject to}\\ \begin{array}{c} \alpha_{i}\geq 0,\\ \sum_{i=1}^{m}\alpha_{i}y_{i}=0, \end{array} \end{array} \end{array}$$

where *α* corresponds to the weights of observation samples. The observation samples with nonzero weights represent support vectors. Consequently, the weights of features or voxels are calculated as

(4) $$\begin{array}{c} \omega =\sum_{i=1}^{m}\alpha_{i}y_{i}x_{i}. \end{array}$$

To evaluate the contribution of each feature, the weight *ω* is ranked based on its squared value (*ω*_*i*_)^2^. Finally, the lowest ranking feature is removed from feature sets *F*:

(5) $$\begin{array}{c} F=F-F_{\omega \left[\text{Lowest Ranking}\right]}. \end{array}$$

**Figure 2:**
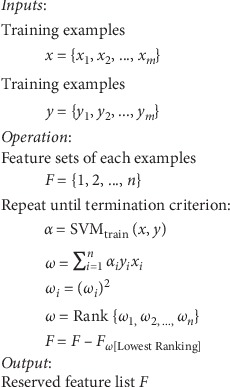
Flow diagram for recursive feature elimination. The entire flow consists of three phases: obtaining the weights of the observation samples *α*, calculating the weight of each feature and ranking based on the weight, and removing the lowest ranking feature from the feature set.

Subsequently, the above process is iterated until a termination criterion is reached or until the feature set *F* is empty. Then, each feature corresponds to a weight, which expresses the importance of the feature. Finally, a user-defined ratio (e.g., 2%) is applied to remove the lower ranking features. The RFE process is shown in Figure 2.

### 2.5. Performance Evaluation

To assess the performance of the proposed method robustly, leave-one-out cross validation (LOO) was applied in this study. Suppose there are *N* samples of SZ patients and *M* samples of NCs; of them, *N*_1_ samples of SZ patients and *M*_1_ samples of NCs are correctly classified. Five performance measures (accuracy, sensitivity, specificity, *F*2 measure, and *G* mean) were used to evaluate the performance:

(6) $$\begin{array}{c} \text{accuracy}\left(\text{ACC}\right)=\frac{\text{TP}+\text{TN}}{\text{TP}+\text{FN}+\text{TN}+\text{FP}}, \end{array}$$

(7) $$\begin{array}{c} \text{sensitivity}\left(\text{SN}\right)=\frac{\text{TP}}{\text{TP}+\text{FN}}, \end{array}$$

(8) $$\begin{array}{c} \text{specificity}\left(\text{SP}\right)=\frac{\text{TN}}{\text{TN}+\text{FP}}, \end{array}$$

(9) $$\begin{array}{c} \text{geometric mean}\left(\text{GM}\right)=\sqrt{\frac{\text{TP}\times \text{TN}}{\left(\text{TP}+\text{FN}\right)\times \left(\text{TN}+\text{FP}\right)}}, \end{array}$$

(10) $$\begin{array}{c} \text{Dice measure}\left(\text{DM}\right)=\frac{2}{\left(\text{TP}+\text{FN}\right)/\text{TP}+\left(\text{TP}+\text{FP}\right)/\text{TP}}, \end{array}$$

(11) $$\begin{array}{c} F2\text{ measure}\left(\text{F}2\text{M}\right)=\frac{5}{4\left(\text{TP}+\text{FN}\right)/\text{TP}+\left(\text{TP}+\text{FP}\right)/\text{TP}}, \end{array}$$

where TP=(*N*_1_/*N*), TN=(*M*_1_/*M*), FN=((*N* − *N*_1_)/*N*), and FP=((*M* − *M*_1_)/*M*). SN represents the proportion of SZ patients predicted correctly, and SP represents the proportion of normal controls predicted correctly. GM, DM, and F2M are defined as the harmonic mean of SN and precision (TP/(TP+FP)), of which GM and DM are computed with the same weights of SN and precision and F2M is computed with higher weights of SN.

## 3. Results

To verify the performance of the coarse-to-fine feature selection proposed in this study, we compared it with six other machine learning methods: (1) directly using SVM to classify (SVM), (2) using two-sample *t*-tests to select features and SVM to classify (2T + SVM), (3) using RFE to select features and SVM to classify (RFE + SVM), (4) using principal component analysis (PCA) and SVM to classify (PCA + SVM), (5) using independent component analysis (ICA) and SVM to classify (ICA + SVM), and (6) using tree-based feature selection and SVM to classify (TBFS + SVM). Furthermore, to test the performance of the proposed machine learning framework, we compared it with other frameworks that select features roughly based on 2T, perform PCA (ICA, TBFS), and then apply SVM for classification (2T + PCA + SVM, 2T + ICA + SVM, 2T + TBFS + SVM). Finally, we analyzed the biomarkers of SZ using coarse-to-fine feature selection.

### 3.1. Parameter Setting

A linear SVM was applied in this study, and previous studies [52, 53] proved that the linear SVM works better on small sample datasets. The value of the penalty coefficient “C” was set to 1.0 because many experimental tests have shown that a value of 1.0 can obtain a satisfactory discrimination performance.

The number of retained features has a significant impact on the results when using RFE. We tested the effect of different numbers of retained features on the results. The experimental results showed that retaining 40% and 14% of the voxels for GM and WM, respectively, and using an elimination ratio of voxels each round of 5% achieved the best performance.

### 3.2. Two-Sample *t*-Tests

**Figure 3:**
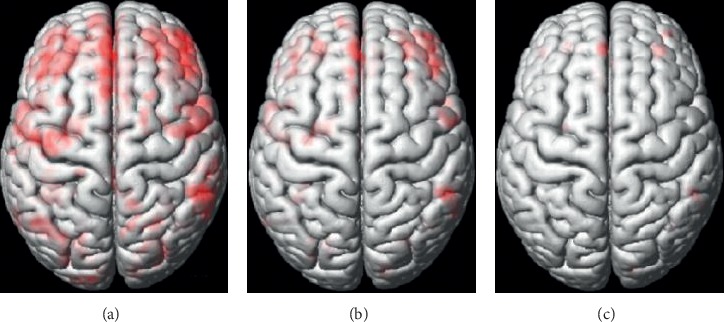
Three two-sample *t*-test maps with different *P* values in GM. The red brain region represents the differential brain region between SZ patients and NCs. As the *P* value becomes stricter (smaller), the differential brain region becomes smaller. (a)*P* < 0.05. (b)*P* < 0.01. (c)*P* < 0.001.

**Figure 4:**
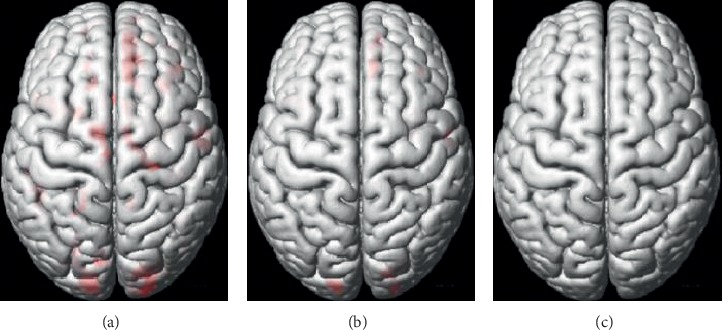
Three two-sample *t*-test maps with different *P* values in WM. As the *P* value becomes smaller, the changes in WM are similar to those in GM. However, compared with GM, the differential WM brain regions between SZ patients and NCs are relatively small. (a)*P* < 0.05. (b)*P* < 0.01. (c)*P* < 0.001.

To test the performance of different *P* values, two-sample *t*-tests were performed with three different *P* values (<0.05 [57–59], <0.01 [60], and <0.001 [61]). The cluster-size value was set to 50 [62–65], and three differentiated tissue maps of GM and WM were obtained and are shown in Figures 3 and 4. From the figures, we can see that as the *P* values decrease, the region selected (red parts) becomes smaller. Consequently, the smaller the *P* value, the more the information being filtered, which may result in the removal of useful features. Therefore, the *P* < 0.05 criterion was adopted in our coarse-to-fine feature selection algorithm.

### 3.3. Classification Performance

The results of different methods based on GM and WM are shown in Tables 2 and 3, respectively.

From Table 2, we can see that feature selection can achieve a better classification performance than using SVM directly for GM. When using only one feature selection algorithm, such as 2T + SVM, RFE + SVM, or PCA + SVM, RFE + SVM can achieve the best performance, which shows that RFE has a better feature selection ability than other methods. When the coarse-to-fine approach is used, such as 2T + RFE + SVM, 2T + PCA + SVM achieves the best performance and reaches an accuracy of 79.81%. The proposed 2T + RFE + SVM method achieves a similar performance (accuracy of 79.62%).

From Table 3, when only one feature selection algorithm is used for WM, all five feature selection methods (2T + SVM, RFE + SVM, PCA + SVM, ICA + SVM, and TBFS-SVM) have a better classification performance than using SVM directly. However, the performances of RFE + SVM, PCA + SVM, ICA + SVM, and TBFS + SVM are worse than that of 2T + SVM for WM. The reason for this result may be that WM has more redundant features, and RFE, PCA, and other methods did not identify useful features from many irrelevant voxels. In contrast, as a traditional statistical analysis, it is easy to explore significant differences based on prior knowledge about different groups. Four coarse-to-fine frameworks perform better than using single feature selection, and the proposed 2T + RFE + SVM method achieves the best performance and reaches an accuracy of over 85% for WM.

**Figure 5:**
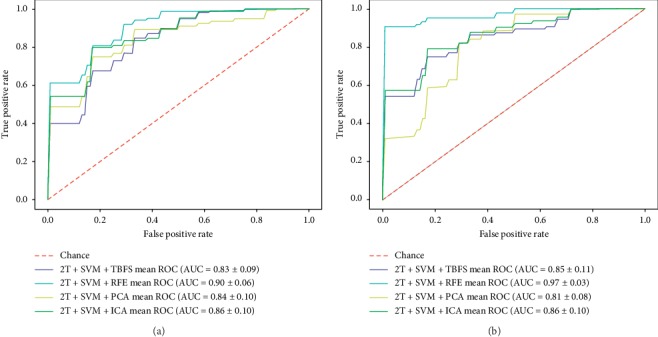
ROC curves using four coarse-to-fine ML frameworks. The ranked list of AUCs is 2T + RFE + SVM, 2T + ICA + SVM, 2T + PCA + SVM, and 2T + TBFS + SVM. The proposed framework, 2T + RFE + SVM, has the best AUC of over 97%. (a) GM. (b) WM.

According to the above experiments, we can see that coarse-to-fine feature selection selects more discriminative features. Using the same coarse-to-fine machine learning framework, RFE can achieve a better performance than the others, and the receiver-operating characteristic (ROC) curves of WM and GM with the proposed ML framework are shown in Figure 5. The AUC represents the performance of the classification experiment. The larger the AUC, the better the performance. It can be seen that the AUC for WM is better than that for GM.

### 3.4. Identification of Abnormal Brain Regions

**Figure 6:**
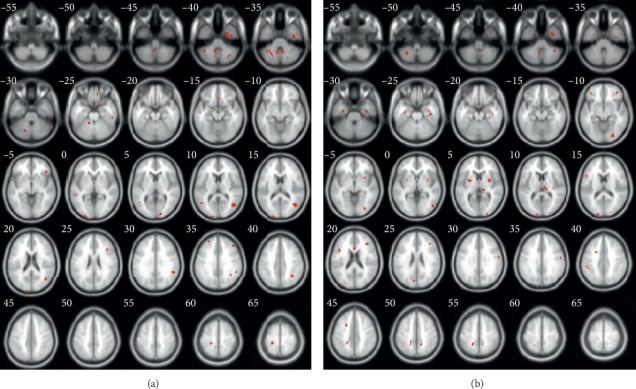
Abnormal brain regions located by our method. The selected feature is shown as a cross section of the brain, which is scanned every 5 mm, and the red brain regions are selected regions. Each discriminative brain region contains more than 50 voxels and has a *P* value <0.05. (a) GM. (b) WM.

The discriminative brain regions (biomarkers) of GM and WM selected by the proposed method are illustrated in Figure 6. We selected clusters as biomarkers when the cluster size was ≥50. For GM, 14 brain regions were detected. For WM, 24 brain regions were detected. Detailed brain biomarkers are shown in Tables 4 and 5. From Table 4, we can see that the GM brain regions that were detected included the cerebellum, fusiform gyrus, temporal lobe, occipital lobe, frontal lobe, right supramarginal gyrus, angular gyrus, and postcentral gyrus. From Table 5, we found that the WM brain regions that were detected included the cerebellum, fusiform gyrus, temporal lobe, occipital lobe, frontal lobe, lentiform nucleus, thalamus, corpus callosum, cuneus, subgyral, and postcentral gyrus. The selected abnormal brain regions were similar in GM and WM. This finding shows that SZ can cause changes in specific brain regions, and these regions are also considered SZ biomarkers.

## 4. Discussion

Previous group-level statistical analysis of neuroimaging data uncovered some neuroanatomical and functional differences between SZ patients and NCs [66–68]. Nevertheless, those findings had limited clinical applications. Machine learning can be adopted for single subject prediction and has shown significant potential for disease diagnosis [69–72] at the individual level.

To verify the performance of the proposed method, we performed the following experiments: (1) We used the SVM directly on smoothed voxels. (2) We used single feature selection and SVM, such as 2T + SVM, RFE + SVM, PCA + SVM, ICA + SVM, and TBFS + SVM. (3) We used coarse-to-fine feature selection methods for classification, such as 2T + PCA + SVM, 2T + ICA + SVM, 2T + TBFS + SVM, and 2T + RFE + SVM. The experiments illustrated the following: (1) SVM classification with feature selection is better than direct SVM classification, which means that the feature selection method can discard redundant features and extract useful information. (2) Among the single feature selection frameworks, 2T + SVM achieved good results for WM, but 2T + SVM required prior knowledge about the groups, which may lead to the poor generalizability of the learning model. Furthermore, using the same framework (RFE + SVM, PCA + SVM, etc. vs. 2T + RFE + SVM, 2T + PCA + SVM, etc.), the frameworks for RFE were synthetically better, which indicated that the features extracted by RFE were more efficient. (3) The performance of the coarse-to-fine framework was better than that of single feature selection methods, which indicates that selecting features hierarchically can filter features more effectively.

Structural abnormalities have been repeatedly demonstrated in SZ patients compared to NCs in previous MRI studies [73–75]. However, most research has focused on DTI to analyze structural changes in SZ and has rarely found structural differences on sMRI. In the current study, the GM and WM features selected by the proposed method provided discriminatory information about anatomically abnormal patterns in schizophrenia. The proposed method in the current study revealed sensitive and accurate information about anatomically abnormal patterns in the frontal lobe, postcentral gyrus, corpus callosum, and cuneus, especially in the thalamus, fusiform gyrus, temporal lobe, and cerebellum. These identified abnormal biomarkers were consistent with those found in the literature [8, 76–79]. Some brain regions were found in both GM and WM, including the cerebellum, fusiform gyrus, temporal lobe, occipital lobe, and frontal lobe, which showed that SZ could indeed cause structural changes in these brain regions. Many previous studies have reported that the concentrations of some substances (such as dopamine) change in the cingulate cortex and amygdale in SZ, but we did not detect structural changes in these areas. The possible reason is that changes in the dopamine concentration do not cause structural abnormalities.

This study has several limitations. First, a small sample size is a common pitfall in most similar studies. To improve the generalizability and clinical applicability of machine learning methods, more samples need to be collected in future studies. Second, only sMRI data were investigated. Multimodal data, such as fMRI, DTI, and brain connectivity data, remain to be explored to provide either complementary or additional information for accurate recognition and lateralization in SZ. Finally, we identified the discriminative regions based on the AAL, BA, and JHU atlas, with the potential drawback that some atlas areas might be too large or unspecific to detect group differences.

## 5. Conclusion

In this study, a coarse-to-fine ML framework was investigated to differentiate SZ patients from NCs and to detect biomarkers of SZ using sMRI. The experiments demonstrated that feature selection algorithms can effectively improve the classification performance. The use of coarse-to-fine feature selection extracted more effective information and significantly improved the classification accuracy. The experiments also indicated that the classification performance of WM was significantly better than that of GM. Therefore, it can be concluded that SZ has a greater impact on WM. This conclusion is consistent with previous findings. Furthermore, the proposed coarse-to-fine feature selection effectively located abnormal brain regions, which provides a helpful aid for the clinical diagnosis of SZ. As a universal method, the proposed framework can be extended to diagnose other diseases.

## Figures and Tables

**Table 1:** Demographic information for patients with SZ and NCs.

	SZ	NC
Gender (M/F)	27/7	23/11
Age	36.85 ± 10.91	39.53 ± 10.59

Note: SZ, schizophrenic; NC, normal control; M, male; F, female.

**Table 2:** Classification performance of different methods based on GM.

Method	ACC	SN	SP	GM	DM	F2M
SVM	0.6277	0.6923	0.5711	0.6288	0.6167	0.6339
2T + SVM	0.6829	0.7226	0.6431	0.6817	0.6741	0.6812
RFE + SVM	0.7326	0.8427	0.6533	0.7420	0.7321	0.7699
PCA + SVM	0.7057	0.7712	0.6521	0.7092	0.7029	0.7103
ICA + SVM	0.6806	0.7114	0.6862	0.6987	0.6571	0.6968
TBFS + SVM	0.7118	0.8200	0.6267	0.7169	0.7155	0.7327
2T + PCA + SVM	0.7981	0.8193	0.7704	0.7945	0.7896	0.7922
2T + ICA + SVM	0.7504	0.7727	0.7111	0.7413	0.7359	0.7394
2T + TBFS + SVM	0.7794	0.8009	0.7484	0.7742	0.7658	0.7701
*Ours*	*0.7962*	*0.8142*	*0.7777*	*0.7957*	*0.7851*	*0.7896*

**Table 3:** Classification performance of different methods based on WM.

Method	ACC	SN	SP	GM	DM	F2M
SVM	0.5689	0.5674	0.5716	0.5695	0.5621	0.5665
2T + SVM	0.7832	0.7818	0.7871	0.7844	0.7798	0.7788
RFE + SVM	0.6427	0.6700	0.6300	0.6497	0.6418	0.6469
PCA + SVM	0.6172	0.5968	0.6370	0.6166	0.6101	0.5996
ICA + SVM	0.5774	0.5796	0.5830	0.5813	0.5721	0.5740
TBFS + SVM	0.5879	0.5547	0.6211	0.5867	0.5662	0.5593
2T + PCA + SVM	0.8372	0.8474	0.8323	0.8398	0.8338	0.8375
2T + ICA + SVM	0.7992	0.7992	0.8037	0.8014	0.7952	0.7978
2T + TBFS + SVM	0.8197	0.7926	0.8351	0.8136	0.8080	0.7965
*Ours*	*0.8527*	*0.8587*	*0.8508*	*0.8547*	*0.8497*	*0.8532*

**Table 4:** Abnormal brain regions (biomarkers) of GM.

Location	Region of interest (ROI)		Size of cluster	MNI coordinates			*P* value
	AAL	BA		*X*	*Y*	*Z*	
lCbe9	105	5	167	−6	−55.5	−45	0.05
rCbe9	106	—	88	9	−54	−45	0.05
FFG.R	56	20	251	31.5	−8	−41.5	0.05
rCbeCru1	92	—	96	34.5	−63	−39	0.05
lCbeCrul	91	—	168	−36	−53.5	−37	0.05
lCbe6	99	—	87	−36	−52	−41.5	0.05
lCbe4-5	97	—	61	−18	−42	−28.5	0.05
ITG.R	90	20	63	55.5	−30	−30	0.05
MOG.L	51	17	127	−26.5	−96.5	0	0.05
SOG.L	49	17	62	−22	−96.5	15.5	0.05
MTG.R	86	37	311	45.5	−66	14.5	0.05
MTG.L	85	37	69	−48	−61.5	4.5	0.05
IFGtriang.R	14	48	60	40.5	19.5	21	0.05
SMG.R	64	48	77	51	−48	27	0.05
ANG.R	66	40	69	36	−56	37	0.05
PoCG.L	57	3	87	−21	−34.5	58.5	0.05

**Table 5:** Abnormal brain regions (biomarkers) of WM.

Location	Region of interest (ROI)			Size of cluster	MNI coordinates			*P* value
	AAL	BA	JHU		*X*	*Y*	*Z*	
lCbe8	103	—	—	76	−30	−58.5	−52.5	0.05
rCbe8	104	—	—	79	37.5	−50	−40.5	0.05
rCbe9	106	—	—	53	7.5	−50	−45	0.05
FFG.R	56	20	—	256	36	−12.5	−34.5	0.05
ITG.R	90	36	—	78	32.5	−1	−41.5	0.05
lCbe9	105	—	—	67	−9	−57	−43.5	0.05
FFG.L	55	20	—	94	−32.5	−10.5	−30	0.05
FFG.R	56	18	—	66	30	−81.5	−10	0.05
ORBinf.L	15	47	—	61	−37.5	39	−15	0.05
PUT.R	74	48	33	136	31.5	9	−6	0.05
SOG.R	50	17	—	72	18	−102	−4.5	0.05
THA.R	78	—	—	239	16.5	−30	−1.5	0.05
PUT.L	73	48	34	127	−28.5	0	4	0.05
SOG.L	49	17	—	92	−12	−100	9	0.05
IFGoperc.L	11	48	—	80	−48	13.5	15	0.05
CcSum	—	—	4	73	1.5	15	16.5	0.05
MFG.R	8	48	—	75	37.5	34	21.5	0.05
CUN.L	45	18	—	70	−7.5	−79.5	22.5	0.05
PoCG.R	58	43	—	86	63	−13.5	24	0.05
IPL.L	61	40	—	70	−46.5	−39	34.5	0.05
Subgyral	—	—	26	160	−21	6	37.5	0.05
PoCG.L	57	—	—	53	−19.5	−43.5	43.5	0.05
PCUN.R	68	—	—	54	13.5	−49.5	48	0.05

## Data Availability

The datasets used in the current study can be obtained from the Centers for Biomedical Research Excellence (COBRE) at http://fcon_1000.projects.nitrc.org/indi/retro/cobre.html.
